# Hallucinations and auditory-verbal signal detection studies: A meta-analytic review

**DOI:** 10.1017/S0033291726105339

**Published:** 2026-08-10

**Authors:** Ruth Medcalf-Bell, Peter Moseley, Emma Barkus

**Affiliations:** Northumbria University, UK

**Keywords:** hallucinations, meta-analysis, signal detection

## Abstract

Auditory-verbal signal detection tasks (AV-SDTs) are used to study hallucinatory experiences (HEs) in clinical and nonclinical groups, assessing perceptual decision making under ambiguous auditory conditions. Existing findings suggest that HEs are associated with response biases. However, effects sizes and subgroup differences remain unclear. This meta-analysis synthesized findings to quantify associations between HEs and AV-SDT performance, testing differences within clinical and nonclinical populations. Systematic searches identified studies published between January 1985 and April 2025. Reference lists were screened and authors contacted. Eligible studies examined associations between hallucinations or hallucination-proneness and AV-SDT performance using between-group or correlational designs. Data comprising 1,391 participants were pooled from 5 clinical (210 patients), and 10 non-clinical (1,181 participants) studies. Moderate-to-large effects were found between HEs and response bias across aggregate (*g* = −0.56 [95% CI: −0.84, −0.28, *p* = .001]), clinical (*g* = −0.88 [95% CI: −1.58, −0.17, *p* = .026]), and nonclinical (*g* = −0.43 [95% CI: −0.74, −0.11, *p* = .013]) subgroup models. Perceptual sensitivity was reduced in nonclinical (*g* = −0.17 [95% CI: −0.28, −0.07, *p* = .004]), but not clinical (*g* = 0.37 [95% CI: −0.00, 0.75, *p* = .050]), hallucinating samples. HEs are consistently associated with a liberal response bias across populations. HEs in nonclinical samples were linked to reduced perceptual sensitivity although this was not supported in clinical groups, potentially due to limited data. Findings highlight methodological gaps in AV-SDT research, including variability in design and hallucination assessment, underscoring the need for standardized approaches to improve generalizability across studies.

## Introduction

Hallucinatory phenomena are involuntary sensory experiences occurring without external stimuli (Nazimek, Hunter, & Woodruff, 2012). Auditory verbal hallucinations (AVHs), the perceptual experience of hearing speech in the absence of an external source (Moseley, Fernyhough, & Ellison, 2013), affect 60%–80% of people with schizophrenia (Waters et al., 2012). However, hallucinatory experiences (HEs) are transdiagnostic (Rollins et al., 2019; Waters & Fernyhough, 2017), occurring in a minority of the general population without psychiatric diagnosis (Hill & Linden, 2013; Linszen et al., 2022), of whom 39% require no intervention (Romme & Escher, 1989). A dimensional model has been proposed, positioning hallucinations along a continuum (Os et al., 2009; van Os, Hanssen, Bijl, & Ravelli, 2000) from clinical to nonclinical experiences, differing in severity, distress, and impairment (Waters & Fernyhough, 2019).

### Perception and top-down processes

Contemporary cognitive models argue perception does not reflect reality; rather it is shaped by top-down processes, modulated by arousal, attention, and prior beliefs (Montemayor & Haladjian, 2017; Powers, Kelley, & Corlett, 2016). Reliance on top-down processes may increase the likelihood perceptual judgments are driven by cognitive biases rather than environmental evidence (Stokes, 2012; Vetter & Newen, 2014). Overreliance on these processes, especially when influenced by preexisting cognitive biases, increases susceptibility to perceptual errors, such as hallucinations (Rodriguez-Sanchez, Oloye, Martin, & Hauke, 2023). Observing responses to ambiguous auditory environments is one way to study such susceptibility, and auditory-verbal signal detection tasks (AV-SDTs) provide a controlled, well-established method for examining perceptual decision making.

In AV-SDTs, participants judge whether a near-auditory-threshold voice is present within background noise, producing four key parameters: *hit rate* – proportion of voice-present trials correctly detected; *false alarm rate* – proportion of noise-only trials incorrectly judged as containing a voice; these parameters are then used to calculate *perceptual sensitivity* – the ability to distinguish between voice-present and voice-absent trials; and *response bias* – the propensity to say ‘voice present’. These standardized metrics are a strength of the AV-SDT paradigm, allowing direct comparability of outcomes across studies.

AV-SDTs have been widely used to investigate mechanisms underlying hallucinations; researchers suggest these tasks varyingly measure reality discrimination, source monitoring, inner speech, or externalizing bias (Anderson, Hartley, & Bucci, 2021; Barkus et al., 2007; Bentall & Slade, 1985a; Li et al., 2002). Despite variation in interpretation, these all fall within top-down perceptual processing frameworks, with higher-order cognitive mechanisms shaping the integration of sensory input.

Hallucinations research using AV-SDTs suggests clinical hallucinations are associated with a liberal response bias (i.e. an increased likelihood for patients to indicate they hear a voice in task trials, regardless of whether a voice signal is presented) (Bentall & Slade, 1985a; Chhabra et al., 2016). In contrast, findings for nonclinical samples, and perceptual sensitivity, remain mixed (Laloyaux et al., 2022; Li et al., 2003; Vercammen, de Haan, & Aleman, 2008). A decade-old meta-analysis has previously examined the link between hallucinations and AV-SDTs (Brookwell, Bentall, & Varese, 2013); however, it considered associations between externalizing bias and performance across multiple tasks, including an AV-SDT and two additional paradigms. Consequently, its focus and comparisons differ from the present meta-analysis. Since then, numerous additional studies have been conducted, including one based on a large dataset (Moseley et al., 2021), warranting an updated synthesis. The present meta-analysis provides a contemporary, quantitative review specifically to synthesize the findings from AV-SDTs for all hallucination research since 1985.

Focusing specifically on AV-SDTs allows for rigorous assessment of consistency and reliability across studies, clarifying whether observed effects reflect robust phenomena rather than task-specific variability. This is particularly important given reported differences between clinical and nonclinical hallucination-prone populations (Barkus et al., 2007; Bentall & Slade, 1985a; Bristow et al., 2014; Moseley et al., 2022; Rankin & O’Carroll, 1995), contributing to the debates surrounding dimensional versus categorical views of psychosis. Accordingly, subgroup analyses for clinical and nonclinical populations will be conducted. Note, here, we use the terms ‘hallucination-prone’ or ‘nonclinical hallucinations’ to refer to nonclinical studies assessing hallucinations using self-report questionnaires such as the Launay–Slade hallucination scale (LSHS) (Launay & Slade, 1981), and the phrase ‘hallucinating versus non-hallucinating’ participants for clinical populations. This meta-analysis has two main aims: (i) to investigate whether there is an association between the presence of hallucinations in people with a psychiatric diagnosis and performance on AV-SDTs; and (ii) to investigate whether there is an association between reports of HEs in nonclinical populations and performance on AV-SDTs. While previous literature suggests liberal response biases are associated with hallucinations, due to the anticipated heterogeneity of existing findings and limited prior synthesis within this area, this meta-analysis has an exploratory aim. No specific *a priori* hypotheses were formulated.

## Method

### Open science/preregistration

The current review followed Moher et al.’s (2009) Preferred Reporting Items for Systematic Reviews and Meta-Analyses (PRISMA) guidelines. The study protocol was registered in June 2022 (PROSPERO: CRD 42022342694). The supporting documents and Supplementary Materials were accessed via the Open Science Framework: https://osf.io/mt9vu/overview.

### Search criteria

**Figure 1.**
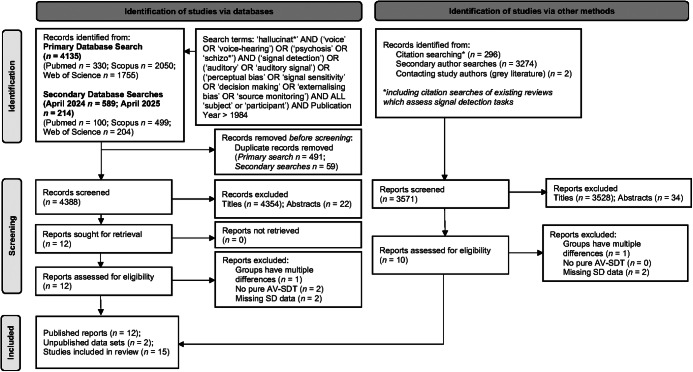
PRISMA flow diagram of database and other methods searches. *Note:* The following eight studies met initial inclusion criteria but were omitted from synthesis after full texts were screened due to lack of an appropriate control group (e.g. comparison between clinical hallucinators and healthy nonclinical, non–voice-hearing controls) (Chhabra et al., 2016, 2022; Moseley et al., 2022 [study 1]); the inclusion of other confounding variables within the signal detection analysis (i.e. hallucinations *and* intrusive thoughts (Smailes, Meins, & Fernyhough, 2014); multiple differences in AV-SDT design (e.g. where tasks required word recognition) (Moseley, Smailes, Ellison, & Fernyhough, 2016; Vercammen et al., 2008); and studies did not report specific signal detection outcomes (Horga, Schatz, Abi-Dargham, & Peterson, 2014; Li et al., 2003). Figure 1. long description.

A database search was conducted on SCOPUS, Web of Science, and PubMed for reports available between January 1985 and April 2025. For a summary see Figure 1 (see Supplementary Materials for full search strategy). Follow-up searches in SCOPUS identified additional studies through author searches and reference screening. Further, first and last authors of all included studies were contacted to identify any relevant published or unpublished work for inclusion.

Studies were eligible if they:

- were written in English;
- examined the relationship between hallucinations or hallucination-proneness and signal detection using AV-SDTs;
- used between-group or correlational designs; and,
- in the case of between-group designs, clearly compared hallucination-prone and appropriate nonprone groups (e.g. comparisons of psychosis patients with versus without hallucinations were eligible, but comparisons of patients with hallucinations versus nonpatients without hallucinations were not eligible).

AV-SDTs were defined as tasks assessing signal detection accuracy, discriminating the presence or absence of a signal within auditory noise, via (i) perceptual sensitivity (i.e. *d′ or P*(*A*)), (ii) response bias (i.e. β, β” or *C*), and, where available, (iii) false alarm and hit rates.

Studies were excluded if they (i) were not pure AV-SDTs (e.g. included additional task demands); (ii) lacked essential data (i.e. none of the following parameters were reported: false alarm and/or hit rates, perceptual sensitivity (*d′ or P*(*A*)), or response bias (β, β” or *C*); (iii) did not explicitly assess hallucinations; (iv) were qualitative, descriptive, non–peer reviewed, conference abstracts, reviews, reanalyses, case studies, or theoretical/philosophical papers; or (iv) were duplicates. Two deviations from preregistration were applied: studies were excluded if (i) hallucinations were not ‘organic’ in nature (i.e. drug, sleep deprivation or illness induced); and (ii) if the signal was not verbal (i.e. not spoken voice).

Titles and abstracts were screened in Rayyan (Ouzzani, Hammady, Fedorowicz, & Elmagarmid, 2016) by blinded reviewers (RMB, EB), with full texts retrieved for eligible or uncertain studies. Disagreements were resolved with a third reviewer (PM). Data extraction was performed independently and in duplicate (RMB all studies; EB and PM splitting duplicates). A standardized form recorded study/participant characteristics, hallucinatory measures, AV-SDT design, and statistics for effect size calculation.

### Effect size computation and integration method

Analyses were conducted in R (Meta v7.0–0; Metafor v4.6–0). Generative AI (ChatGPT; OpenAI, San Francisco, CA) was used solely to assist with debugging R scripts and was not used to generate analytic decisions, interpret findings, or to write or revise the manuscript. Hedges’ *g* (Lin & Aloe, 2021) was used for effect sizes, computed with the *effsize* package v0.8.1 from sample sizes, means and SDs (group studies), or correlation coefficients (correlational studies). Interpretation followed Cohen’s (1992) thresholds (small ≤0.20; medium ≤0.50; large ≤0.80). Prediction intervals (PIs) were calculated using the *t*-distribution (Higgins, Thompson, & Spiegelhalter, 2009). Significance was set at *p* < .05. For group designs, only hallucinating versus nonhallucinating contrasts were analyzed in clinical samples, and low versus high hallucination-prone contrasts in nonclinical samples.

Given expected heterogeneity, random-effects models were applied, with Hartung–Knapp adjustments for pooled confidence intervals (Hartung & Knapp, 2001). Separate summary effects were estimated for hit rate, false alarm rate, perceptual sensitivity (*d*′ or *P*(A)), and response bias (*β* or *c*), followed by subgroup analyses for clinical versus nonclinical samples.

Heterogeneity was assessed using the *Q*-test, *I* ^2^ (Higgins, Thompson, Deeks, & Altman, 2003), and *τ* ^2^ via restricted maximum likelihood. Publication bias was evaluated using funnel plots and Egger’s test (Egger, Davey Smith, Schneider, & Minder, 1997), with Duval–Tweedie Trim and Fill (Duval & Tweedie, 2000) applied where bias was evident. Influence was examined by influence diagnostics and, if warranted, ‘leave-one-out’ analyses were used.

### Quality assessment

Study quality was assessed using the appraisal tool for cross-sectional studies (AXIS) (Downes, Brennan, Williams, & Dean, 2016), which was selected for its suitability in addressing the methodological diversity of observational designs (Losilla, Oliveras, Marin-Garcia, & Vives, 2018; Ma et al., 2020). AXIS comprises 20 items across reporting quality (7), study design (7), and risk of bias (6). All included studies were appraised by RMB (see Supplementary Materials).

## Results

Initial database searches were conducted on July 19, 2022 and updated on April 24, 2024 and April 15, 2025 to capture newly published studies. Figure 1 summarizes the search and selection process. Corresponding authors were contacted to request unpublished data, clarify methods, or supply missing information essential for this meta-analysis. This yielded two unpublished datasets (Alderson-Day (2019); Bell et al., (unpublished)) and additional details for two previously identified studies (Barkus et al. (2007) (In the original study participants were subdivided into three groups based on high, around the mean or low for both ‘positive’ schizotypy scores (measured by Oxford Liverpool Inventory of Feelings and Experiences (Mason, Claridge, & Jackson, 1995)) and reported HEs (measured by LSHS (Launay & Slade, 1981)). Study data were requested to perform correlational analysis. The decision to treat data as correlational (as opposed to group) was based on the value of retaining all data from the original study (i.e. retaining the mean group). Only results derived from the LSHS were included, to capture hallucination-proneness and be consistent with other studies in the review); and Smailes, Meins, and Fernyhough (2015) (In the original study, effect sizes for the association between HEs and AV-SDT performance were not reported)). Abstract screening agreement between the two reviewers was almost perfect, *κ* = .92 (99.96% agreement).

Following full-text screening, 22 records met the initial inclusion criteria outlined above. However, several records were omitted from data synthesis. The reasons for the exclusions are detailed in Figure 1. Following these exclusions, a final total of 15 studies were identified for inclusion in this review. Where included studies followed repeated-measure designs, only results taken from the initial run of the task were included. For results using unpublished data, Spearman’s *ρ* was calculated to assess the association between HEs and AV-SDT performance. No data transformations for signal detection outcomes (hit rate, false alarm rate, perceptual sensitivity, and response bias) were applied (all signal detection outcomes were calculated by the corresponding authors (Barkus et al., 2007; Smailes et al., 2015; Alderson-Day, (2019))). Table 1 summarizes the key features of the study design, location, and sample characteristics of included studies. All included studies were in English. Individual study effect sizes are detailed in the Supplementary Materials.

**Table 1.** Characteristics of included studies within the meta-analytical models Table 1. long description.

Author, Year	Country	Study design	Sample	*N*	Mage (SD)	Gender (%female)	Hallucinatory measure^a^	Reported theoretical paradigm supporting the use of signal detection
Alderson-Day (2019)	UK	Correlation	Nonclinical	95	21.37 (4.88)	75.05	Modified LSHS-R^b^	
Alganami, Varese, Wagstaff, and Bentall (2017)	UK	Group High vs low HE	Nonclinical	62	21.06 (3.09)	74.20	LSHS-R (12-item)	Source-monitoring
Barkus et al. (2007)	UK	Correlation	Nonclinical	62	23.95 (8.60)	50	LSHS (12-item)	Inner speech
Barkus et al. (2011)	UK	Group High vs Low UE	Nonclinical	55	–	–	O-Life	Responding biases in perceptual monitoring tasks
Bell et al. (unpublished)	Australia	Group AVH vs no AVH	Clinical	39	–	–	MHS	
Bentall and Slade (1985a)	UK	Group AVH vs no AVH	1. Clinical	20	38.71 (13.14)	15	LSHS-A (12-item)	Reality testing
		Group High vs low HE	2. Nonclinical	20	19.13 (.93)	0	LSHS-A (12-item)	
Bristow et al. (2014)	UK	Group AVH vs no AVH	Clinical	98	36.83 (10.21)	35.28	SAPS	Reality discrimination and externalising perceptual bias
Moseley et al. (2021)	Multi	Correlation	Nonclinical	583	29.40 (10.90)	55.70	CAPS	Top-down processing on speech
Moseley et al. (2022)	UK	Group AVH vs no AVH	Nonclinical	54	58.70 (11.55)	64.81	LSHS-A	Influence of top-down processes
Rankin & O’Carroll, (1995)	UK	Group High vs low HE	Nonclinical	30	25.4 (1.83)	–	LSHS (12-item)	Reality discrimination
Smailes et al. (2015)	UK	Correlation	Nonclinical	153	21.09 (3.48)	85.60	CAPS (Auditory subscale only)	Reality discrimination
Strawson et al. (2022)	UK	Correlation	Clinical HP-correlational	22	33 (10.80)	72.73	BSIS-V	Externalising biases in source monitoring
Varese, Barkus, and Bentall (2011)	UK	Group High vs low HE	Nonclinical	67	21.63 (5.35)	58.21	LSHS-R (12-items)	Reality discrimination
Varese, Barkus, and Bentall (2012)	UK	Group AVH vs no AVH	Clinical	31	46.99 (12.20)	45.16	SCI-PANSS (hallucinations questions)	Reality discrimination

*Note:* HE, hallucinatory experiences; UE, unusual experiences; AVH, auditory verbal hallucination; BSIS-V, brief symptom impact scale-voices (Strawson et al., 2022); CAPS, The Cardiff anomalous perceptions scale (Bell, Halligan, & Ellis, 2006); LSHS, Launay–Slade hallucinations scale (Launay & Slade, 1981; LSHS-A, Launay–Slade hallucinations scale-A Bentall & Slade, 1985b); LSHS-R (Morrison, Wells, & Nothard, 2000); Modified LSHS-R (McCarthy-Jones & Fernyhough, 2011); MHS, multimodal hallucinations schedule (Bell et al., unpublished)); O-Life, Oxford Liverpool inventory of feelings and experiences (Mason et al., 1995; SAPS, scale for the assessment of positive symptoms (Andreasen, 1984); SCI-PANSS, positive and negative symptom scale (Kay, Opler, & Lindenmayer, 1989).

a This is the measure used within studies to either group participants or provide an overall score of hallucination in correlational studies. It is noted that some studies also include secondary measures of hallucination, but these are not reported here.

b 9-item scale, 5-item auditory subscale only.

### Demographic characteristics

Eligible studies comprised a total of 1,391 participants. Based on the available data, 688 were women and 489 were men (these totals do not sum the full sample size as some papers did not specify sex). Mean age, calculated from the available data, was 29.49 years (SD = 8.80). Two studies did not contribute age data (Barkus et al., 2011; Bell et al., (unpublished)), and three studies did not contribute sex data (Barkus et al., 2011; Rankin & O’Carroll, 1995; Bell et al., (unpublished)). It should be noted that Barkus et al. (2011) reported demographic characteristics for the full original sample, the present study included only a subset of participants from that dataset, and corresponding age and sex for that subset could not be determined. In total, 10 studies presented data from nonclinical populations (*n* = 1,181) and 5 studies from patient groups (*n* = 210).

### Auditory-verbal hallucination status

HEs were evaluated using several measures across studies (see Table 1). While the included nonclinical studies used a version of the Launay–Slade hallucination scale (LSHS; Bentall & Slade, 1985b; Launay & Slade, 1981; Morrison et al., 2000; *n* = 8); the Oxford–Liverpool Inventory of Feelings and Experiences (O-Life; Mason et al., 1995; *n* = 1); or the Cardiff anomalous perceptions scale (CAPS) (Bell et al., 2006) (*n* = 2), no two clinical studies used the same methodology of assessing HEs. Furthermore, defining cutoffs for high versus low hallucinators varies across studies; for example, general population studies comparing performance across groups used the LSHS to group participants: either by an arbitrary selection of either the highest versus lowest scorers (Bentall & Slade, 1985a; Rankin & O’Carroll, 1995); an upper and lower tertile split (Alganami et al., 2017; Barkus et al., 2011; Varese et al., 2011); or, simply to quantify participants HEs, after the study cohort had already been screened and grouped by the study researchers (Moseley et al., 2022).

### AV-SDT design

See the Supplementary Materials for details of individual study AV-SDT designs. Notably, several published studies omit details of the task administration and instruction. Several studies incompletely report AV-SDT procedures, including participant instructions, practice trials, and signal frequency information. Furthermore, reporting details of the task design also varied across studies, and details such as the language of the spoken voice and voice duration were underreported. All published studies reported the number of trials included per run (mean = 66.83), ratio of noise to noise-and-signal trials (mean ratio = 44:56), and duration of the noise presentation for each trial (mean duration = 4.46secs).

### HEs and AV-SDT performance

**Figure 2.**
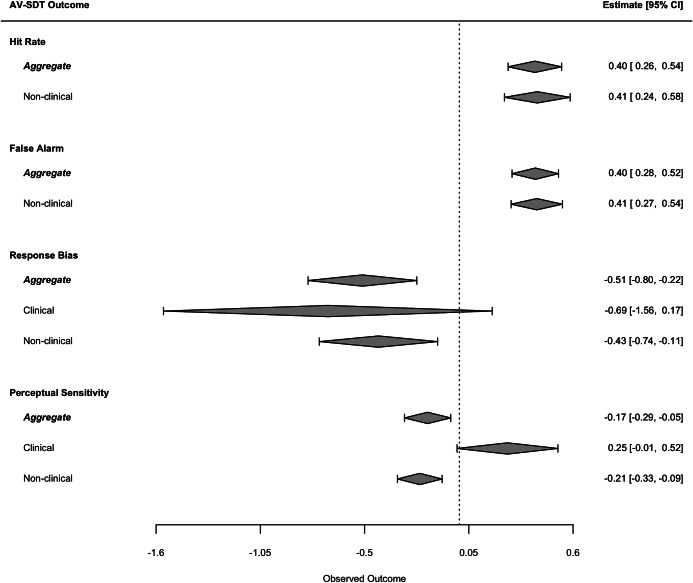
Summary forest plot of the pooled random effects aggregate and subgroup (clinical and nonclinical) analyses for the association between hallucinatory experiences and AV-SDT outcomes. CI, Confidence interval. *Note:* Only aggregate and nonclinical subgroup effects are reported for false alarm and hit rate because, to date, only one clinical study has published these specific findings. Figure 2. long description.

A summary of all analyses for both main and within subgroup associations between HEs and AV-SDT performance is presented in Table 2. Summary forest plots of all study main and subgroups effects are displayed in Figure 2.

**Table 2.** Effect sizes for the aggregated and subgroup (clinical versus nonclinical) analyses, stratified by AV-SDT parameter (hit rate, false alarm rate, perceptual sensitivity, or response bias) Table 2. long description.

Outcome	Model	*k*	*g* ([95% CI], *p*)[Meta-analysis]	*τ* ^2^ (LL, UL)[Meta-analysis]	*I* ^2^ (LL, UL)[Meta-analysis]	*β*₀ (95% CI), *t*(df), *p*[Publication bias]
False alarm rate	Aggregate	8	0.40 ([0.28, 0.52], < .001)	0.01 (0.00, 0.06)	0.0% (0.0%, 67.6%)	0.77 (−0.25, 1.79), *t*(7) = 1.48, *p* = .189
	Clinical	1	–	–	–	–
	Nonclinical	7	0.41 ([0.27, 0.54], < .001)	0.01 (0.00, 0.07)	7.4% (0.0%, 73.0%)	1.08 (−0.01, 2.16), *t*(6) = 1.44, *p* = .110
Hit rate	Aggregate	6	0.40 ([0.26, 0.54], < .001)	0.00 (0.00, 0.28)	17.2% (0.0%, 62.0%)	0.18 (−1.55, 1.9), *t*(5) = 0.20, *p* = .850
	Clinical	1	–	–	–	–
	Nonclinical	5	0.41 ([0.24, 0.58], .003)	0.00 (0.00, 0.27)	23.5% (0.0%, 68.7%)	0.79 (−1.27, 2.85), *t*(4) = 0.75, *p* = .507
Perceptual sensitivity	Aggregate	13	−0.16 ([−0.30, −0.01], .038)	0.01 (0.00, 0.17)	19.9% (0.0%, 57.8%)	0.79 (−1.27, 2.85), *t*(12) = 0.75, *p* = .507
	Clinical	4	0.37 ([−0.00, 0.75], .050)	0.00 (0.00, 0.83)	0.0% (0.0%, 84.7%)	−0.23 (−3.07, 2.62), *t*(3) = −0.16, *p* = .890
	Nonclinical	9	−0.17 ([−0.28, −0.07], .004)	0.00 (0.00, 0.70)	0.0% (0.0%, 64.8%)	−0.36 (−1.38, 0.66), *t*(8) = −0.69, *p* = .513
Response bias	Aggregate	15	−0.56 ([−0.84, −0.28], .001)	0.14 (0.03, 0.60)	62.7% (34.8%, 78.6%)	−1.21 (−2.25, 0.00), *t*(14) = −1.96, *p* = .073
	Clinical	5	−0.88 ([−1.58, −0.17], .026)	0.18 (0.00, 2.25)	51.4% (0.0%, 82.2%)	−2.51 (−7.37, 2.36), *t*(4) = −1.01, *p* = .387
	Nonclinical	10	−0.43 ([−0.74, −0.11], .013)	0.08 (0.01, 0.84)	62.0% (24.5%, 80.9%)	−0.77 (−2.33, 0.79), *t*(9) = −0.97, *p* = .362

*Note: CI, confidence interval; LL, lower limit; UL, upper limit; and df, degrees of freedom.*

Aggregate analyses revealed a significant, positive association between HEs and rates of responding (i.e. hit rate and false alarm rate) during AV-SDT trials. The PI indicates future single studies are unlikely to show negative associations (HEs and false alarm rate g = 0.13 to 0.67; HEs and hit rate g = 0.25 to 0.55).

To date, only one study of clinical populations has detailed participant AV-SDT hit and false alarm rates. Consequently, only subgroup analyses of nonclinical associations are reported for these parameters. These analyses revealed a significant, positive association between nonclinical HEs and both hit rate and false alarm rate during AV-SDT trials. Between-study heterogeneity was low, and the PIs suggest future single studies are unlikely to show negative associations for either outcome (false alarm rate *g* = 0.11 to 0.70; hit rate *g* = 0.20 to 0.62).

Due to convergence issues within the data set, *τ* ^2^ was estimated using the DerSimonian–Laird method for the following associations.

### HEs and perceptual sensitivity

The results show a significant negative association between HEs and perceptual sensitivity in aggregate and nonclinical models, but not in the clinical subgroup. Subgroup effects differed significantly (*Q*(1) = 18.94, *p* < .001). Between*-*study heterogeneity was low. The PIs indicate considerable heterogeneity across future studies. For both the aggregate (g = −0.41 to 0.10) and clinical models (g = −0.29 to 1.03), the intervals include both positive and negative values, implying future studies may plausibly yield effects in either direction. In contrast, the PI for nonclinical studies (g = −0.28 to −0.07) lies entirely below zero, suggesting consistently negative effects.

### HEs and response bias

Results show HEs were negatively associated with response bias in aggregate and subgroup models. Subgroup differences were nonsignificant (*Q*(1) = 2.43, *p* = 0.119). Between study heterogeneity was high and PIs indicate substantial heterogeneity across future studies. For all study models (aggregate [g = −1.40 to 0.27], clinical [g = −2.29 to 0.53], and nonclinical [g = −1.13 to 0.27]), the intervals spanned both negative and positive values, suggesting future studies could plausibly observe effects in either direction.

### Influence and sensitivity assessment

Outliers were revealed in both the aggregate (*n* = 2) and nonclinical subgroup (*n* = 1) models for HEs and response bias. Excluding the study outliers did not significantly influence the aggregate model (omitting Strawson et al. (2022) and Barkus et al. (2007): *k* = 13, *g* = −0.46 (95% CI: −0.60, −0.31), *p* < .001; *τ* ^2^ = 0.01(95% CI: 0.00, 0.35), and *I* ^2^ = 18.9% (95% CI: 0.0%, 57.1%); but it did substantially influence the observed probability of the nonclinical subgroup model (*k* = 9, *g* = −0.44 (95% CI: −0.61, −0.27), *p* < .001; *τ* ^2^ = 0.01(95% CI: 0.00, 0.69), and *I* ^2^ = 31.4% (95% CI = 0.0%, 68.3%). No other outliers were found in any other model.

An additional sensitivity analysis was conducted which examined the potential influence of small-sample effects. Studies with hallucinating-group sample sizes ≤20 were excluded from between-group analyses of HEs and response bias, while correlational studies were retained. Exclusion of these studies, alongside identified outliers (Strawson et al., 2022 and Barkus et al., 2007), substantially influenced the aggregate model (*k* = 8, *g* = −0.39 [95% CI: −0.47, −0.31], *p* < .001; *τ* ^2^ = 0.00 [95% CI: 0.00, 0.02], *I* ^2^ = 0.0% [95% CI: 0.0%, 67.6%)]. The PIs suggest future individual studies are unlikely to demonstrate positive effects. Together, these findings indicate smaller cell size studies contribute to variability in the observed response bias effects although the overall direction of effects remains consistent with the primary analyses.

The results of leave-one-out sensitivity analysis where the omission of a study substantially increased study effects (>10% change) and reduced observed heterogeneity are listed in Supplementary Materials.

### Quality assessment

See the Supplementary Materials for AXIS quality ratings for individual studies. Quality assessment followed recent reporting of AXIS ratings (studies which had a frequency of ‘Yes’ responses of ≥80% were rated as high quality; 60%–80% as moderate quality; and <60% as low quality). Most studies were rated moderate quality (*n* = 7), one was rated high quality, and the remaining studies were rated low quality (*n* = 5). Mean subscale scores across studies showed moderate quality rating of study design (mean score = 5.54) and reporting quality (mean score = 4.31), and low reporting quality of possible biases (mean score = 2.15). Several methodological aspects were consistently underreported across studies. Common weaknesses included inadequate reporting of sample justification, nonresponders, and response-rate bias, which substantially reduced AXIS ratings.

## Discussion

Critically, this meta-analysis shows a liberal response bias during AV-SDTs associated with hallucinations, in both clinical and nonclinical groups. Nonclinical hallucinators also showed increased false alarm and hit rates, although underreporting of response frequencies in clinical studies limits generalization of this effect. Additionally, wide PIs indicate effect size estimates should be interpreted with caution. Additionally, HEs were linked to reduced perceptual sensitivity, but this association was negligible in clinical subgroups.

These results align with research demonstrating an association between overreporting of voices in noise and hallucinations (Barkus et al., 2011; Bentall & Slade, 1985a; Moseley et al., 2021; Varese et al., 2012). Although consistent with a previous meta-analysis by Brookwell et al. (2013), in demonstrating an association between hallucinations or a tendency to hallucinate and response bias, the effect observed here is smaller, likely reflecting more recent datasets with larger sample sizes. Brookwell et al. (2013) interpreted this effect as evidence for an externalizing bias (i.e. hallucinations result from a bias toward misattributing internally generated mental events to an external source), but our review highlights different authors interpreted findings from AV-SDTs as supporting inner speech, reality discrimination, or source monitoring difficulties. While biased AV-SDT performance likely broadly relates to the extent to which an individual relies on top-down processing in response to ambiguous stimuli, further studies are now needed to elucidate precisely if and how this relates to other cognitive processes.

Since findings for response bias were evident in both clinical and nonclinical groups, it suggests a shared perceptual bias, rather than discrete cognitive differences associated with pathological processes (Alderson-Day et al., 2017; de Boer et al., 2019). This result challenges theories proposing psychiatric symptoms impair perceptual judgements beyond that experienced by nonclinical hallucinators (Powers, Mathys, & Corlett, 2017).

The finding of reduced sensitivity in hallucinators implies for diminished discrimination for speech signals, contrasting earlier studies reporting no differences (Alganami et al., 2017; Barkus et al., 2007; Bentall & Slade, 1985a). Yet, this effect was not significant within clinical samples, contrasting Vercammen et al. (2008) who proposed atypical auditory verbal processing common to hallucinators regardless of psychopathology. Furthermore, subgroup differences were significant, potentially indicating different mechanisms of misperception in clinical and nonclinical hallucinators. However, the small number of clinical studies and wide PIs warrant cautious interpretation (Riley, Higgins, & Deeks, 2011; Turner, Bird, & Higgins, 2013). Alternative explanations should be considered, including differences in medication status, particularly antipsychotic treatments, or underlying pathophysiology, which may influence attentional processes and perceptual sensitivity. Consequently, more research, with larger, well-characterized clinical cohorts is needed to disentangle these factors and determine whether reduced perceptual sensitivity is consistently associated with hallucinations in those cohorts.

Unlike previous meta-analyses primarily focusing on response bias and including AV-SDTs with other paradigms, this study compared perceptual sensitivity, response bias, and hit and false alarm rates specifically for AV-SDTs. No evidence of publication or selection bias was found, though large-sample studies (e.g. (Moseley et al., 2021, *n* = 594) disproportionately influenced results, particularly in the non-clinical model (*N* = 1,192).

This review highlights variability and underreporting in both experimental design and results. Notably, between study heterogeneity was moderate to high (Higgins & Thompson, 2002) for the association between hallucinations and AV-SDT response bias (e.g. *I* ^2^ = 62.7%), suggesting observed effects may be attributable to methodological differences, including task design and administration, and variability in participant groups such as hallucination classification, medication status, and illness chronicity.

Quality assessment using AXIS rated most studies as moderate-to-high quality, with approximately 40% rated low mainly due to insufficient reporting of cohort characteristics, nonresponder identification, and measure reliability. Encouragingly, virtually all studies conducted within the last decade show improved reporting standards. This could reflect changes in attitudes from the publishing community to permit more generous word counts, Supplementary Materials being more common, and recognition that higher levels of detail are required in methods sections.

### Limitations

A major limitation of this literature analysis is the way hallucinations are measured, classified, defined (i.e. modality), and cutoffs applied varies across studies. Such variability raises concerns about comparability of ‘hallucinators’, as studies may capture fundamentally different experiences and constructs (Longden et al., 2020). Two specific limitations are as follows: (i) no analyses have examined the influence of measurement choice on outcomes, and (ii) this review only distinguishes between clinical and nonclinical populations when examining signal detection performance and HEs, without further subgrouping within those categories. This focus overlooks important heterogeneity within samples, including symptom levels, and definitions of ‘hallucination-prone’ individuals. Consequently, group-level contrasts may obscure meaningful gradations in hallucinatory experience. These methodological differences may partly account for the heterogeneity observed in associations between reported hallucinatory experiences and both response bias and perceptual sensitivity in the current synthesis. Further research is required to determine how variation in hallucination assessment methods shapes these findings.

Demographic variability may also influence outcomes. Clinical samples were, on average, older (*M* = 38.40, SD = 11.75) than nonclinical samples (*M* = 27.49, SD = 11.89), an expected pattern given the reliance of some nonclinical studies (Alganami et al., 2017; Barkus et al., 2011; Bentall & Slade, 1985a; Smailes et al., 2015; Varese et al., 2011) on student populations. These age differences could contribute to variation between cohorts, as hallucinatory predisposition tends to decline over the lifespan (Shenyan et al., 2025). If perceptual-cognitive characteristics differ systematically across groups, age-related effects could represent a key moderating or distinguishing factor. However, this possibility could not be examined because several included studies did not report participant age, resulting in insufficient data across studies to permit a reliable analysis. Future studies should consider greater demographic matching and consistent reporting to improve comparability across AV-SDT research.

Another limitation of this meta-analysis is the potential for analyses to be underpowered due to the small number of studies and their relatively small sample sizes, which may induce ‘small study effects’ and moderate heterogeneity (Turner et al., 2013). For perceptual sensitivity and response bias, the PIs for model effects cross zero indicating effects in the opposite direction cannot be ruled out (IntHout, Ioannidis, Rovers, & Goeman, 2016). This issue is particularly pronounced in clinical subsamples, where only five studies were available, several of which had small sample sizes (e.g. *n* ≤30 (Bentall & Slade, 1985a,1985b; Varese et al., 2012)). Accordingly, these findings should be interpreted with caution, particularly with respect to their generalizability. More meta-analytical comparisons are needed to distinguish methodological from perceptual differences.

Lastly, limiting inclusion to English-language publications may have excluded relevant studies and introduced potential language bias limiting generalizability across populations.

### Future directions

Thorough reporting of AV-SDT design and outcomes is essential for future development of the task paradigm. Many studies included here lack detail on task parameters (trial numbers, noise-to-signal ratios, stimulus properties), participant instructions, and response frequencies (hit/false alarm rates), hindering replication and interpretation. Reporting these data is important, as optimal decision making (criterion) is affected by task demands (Lynn & Barrett, 2014). Moreover, inconsistent reporting of effect sizes for nonsignificant outcomes risks publication bias (Nair, 2019). Table 3 provides detailed reporting recommendations to improve transparency.

**Table 3.** Recommendations for future reporting of AV-SDTs Table 3. long description.

Identifying AV-SDTs	Inclusion of ‘signal detection’ in study identifying information *(i.e. title or keywords)* Explicit use of terminology for AV-SDTs; specify these are signal detection tasks and what theoretical framework this has been applied to *(i.e. reality monitoring, externalizing bias etc.)*
Task parameters	Specify instructions given to participants *(i.e. whether participants were aware of frequency of noise to signal in noise trials)* Identify features of signal detection task trials *(i.e. include total number of trials; ratio of noise to noise-and-signal trials; duration of individual trials; and details of durations of sound presentation)* Identify study sounds *(i.e. specify what the noise is, what the voice is saying, and what the frequencies or volumes of sound presentation are)*
Testing environment	Identify equipment used and the location of the testing environment
Task outcomes	Reporting of signal detection outcomes *(i.e. include frequencies of hit rate AND false alarm rate AND perceptual sensitivity AND bias)*

The details of design differences across studies are provided in the Supplementary Materials. While such variations (e.g. stimulus type, number of trials, testing environment) may confound findings by influencing participants’ expectations and decision making (Ghiani, Mann, & Brenner, 2024), assessing their impact is beyond the scope of this review. Future research should examine these factors, perhaps incorporating distractor or practice tasks to reduce demand characteristics.

Overall, AV-SDTs offer a valuable paradigm to objectively study hallucination-related misperceptions and their cognitive underpinnings. Confidence in perceptual decisions, noted in previous research (Bentall & Slade, 1985a), was rarely reported here and warrants inclusion in future work to better understand subgroup differences. Simple additions, such as the inclusion of confidence ratings, could enrich study designs and illuminate underlying mechanisms.

## Conclusions

These meta-analyses present evidence for an association between HEs and response bias, revealing a pattern of liberal response behavior observable in both clinical and nonclinical groups, and consistent with a role for overweighted top-down processes in hallucinations. In contrast to previous research, results show that hallucinatory phenomena are associated with a decrease in perceptual sensitivity. This finding seems primarily influenced by data from nonclinical studies, likely reflecting limited clinical studies reporting on perceptual sensitivity (and other signal detection parameters). Consequently, generalizability of this effect to clinical populations remains unclear. Synthesis of the included literature highlights several gaps in reporting, including the possible influence of variation in study designs and differences in the assessment of hallucinations.

## Supporting information

10.1017/S0033291726105339.sm001 Medcalf-Bell et al. supplementary material Medcalf-Bell et al. supplementary material

## Supplementary material

The supplementary material for this article can be found at http://doi.org/10.1017/S0033291726105339.

## Long descriptions

### Figure 1. Long description

The flowchart is organized into three vertical phases: Identification, Screening, and Included.

1. Identification Phase:

* Left Column (Databases): Records identified from Primary Database Search (n = 4135) and Secondary Database Searches (April 2024 n = 589; April 2025 n = 214). A box to the right lists search terms including ‘hallucinat*’, ‘voice-hearing’, and ‘signal detection’. Below this, 550 duplicate records were removed (491 from primary, 59 from secondary).

* Right Column (Other Methods): Records identified from Citation searching (n = 296), Secondary author searches (n = 3274), and Contacting authors (n = 2).

2. Screening Phase:

* Left Column: 4388 records screened, leading to 4376 exclusions (4354 titles, 22 abstracts). 12 reports were sought for retrieval and all 12 were assessed for eligibility. 5 reports were excluded due to group differences (n = 1), no pure A V - S D T (n = 2), or missing S D data (n = 2).

* Right Column: 3571 reports screened, leading to 3562 exclusions (3528 titles, 34 abstracts). 10 reports were assessed for eligibility, with 3 excluded for the same reasons as the left column.

3. Included Phase:

* Arrows from both columns converge at the bottom into a final box: Published reports (n = 12), Unpublished data sets (n = 2), and Studies included in review (n = 15).

Navigate back to Figure 1..

### Table 1. Long description

The table contains nine columns: Author, Year; Country; Study design; Sample; N; M age (S D); Gender (% female); Hallucinatory measure; and Reported theoretical paradigm.

Key entries include:

* Alderson-day (unpublished), UK, Correlation, Nonclinical, N=95, M age 21.37.

* Alganami et al. (2017), UK, Group High vs low H E, Nonclinical, N=62, M age 21.06, Source-monitoring paradigm.

* Barkus et al. (2007), UK, Correlation, Nonclinical, N=62, M age 23.95, Inner speech paradigm.

* Barkus et al. (2011), UK, Group High vs Low U E, Nonclinical, N=55, Responding biases paradigm.

* Bell et al. (unpublished), Australia, Group A V H vs no A V H, Clinical, N=39.

* Bentall and Slade (1985a), UK, two groups: Clinical (N=20, M age 38.71) and Nonclinical (N=20, M age 19.13), Reality testing paradigm.

* Bristow et al. (2014), UK, Group A V H vs no A V H, Clinical, N=98, M age 36.83, Reality discrimination paradigm.

* Moseley et al. (2021), Multi-country, Correlation, Nonclinical, N=583, M age 29.40, Top-down processing paradigm.

* Moseley et al. (2022), UK, Group A V H vs no A V H, Nonclinical, N=54, M age 58.70.

* Rankin and O’Carroll (1995), UK, Group High vs low H E, Nonclinical, N=30, M age 25.4.

* Smailes et al. (2015), UK, Correlation, Nonclinical, N=153, M age 21.09, Reality discrimination paradigm.

* Strawson et al. (2022), UK, Correlation, Clinical, N=22, M age 33, Externalising biases paradigm.

* Varese et al. (2011), UK, Group High vs low H E, Nonclinical, N=67, M age 21.63.

* Varese et al. (2012), UK, Group A V H vs no A V H, Clinical, N=31, M age 46.99.

Hallucinatory measures include L S H S, L S H S dash R, O dash Life, M H S, S A P S, C A P S, B S I S dash V, and S C I dash P A N S S.

Navigate back to Table 1..

### Table 2. Long description

The table consists of seven columns: Outcome, Model, k, g (95% C I, p) [Meta-analysis], tau super 2 (L L, U L) [Meta-analysis], I super 2 (L L, U L) [Meta-analysis], and beta sub 0 (95% C I), t(d f), p [Publication bias].

* False alarm rate: Aggregate (k=8, g=0.40, p < .001); Clinical (k=1, no data); Nonclinical (k=7, g=0.41, p < .001).

* Hit rate: Aggregate (k=6, g=0.40, p < .001); Clinical (k=1, no data); Nonclinical (k=5, g=0.41, p = .003).

* Perceptual sensitivity: Aggregate (k=13, g=-0.16, p = .038); Clinical (k=4, g=0.37, p = .050); Nonclinical (k=9, g=-0.17, p = .004).

* Response bias: Aggregate (k=15, g=-0.56, p = .001); Clinical (k=5, g=-0.88, p = .026); Nonclinical (k=10, g=-0.43, p = .013).

Publication bias for Response bias Aggregate shows beta sub 0 = -1.21, t(14) = -1.96, p = .073. Heterogeneity (I super 2) is highest for Response bias at 62.7% for the aggregate model.

Navigate back to Table 2..

### Figure 2. Long description

The forest plot displays observed outcomes on an x-axis ranging from negative 1.6 to 0.6, with a vertical dashed line at zero.

1. Hit Rate section.

- Aggregate: Diamond centered at 0.40 with a 95% C I of 0.26 to 0.54.

- Non-clinical: Diamond centered at 0.41 with a 95% C I of 0.24 to 0.58.

2. False Alarm section.

- Aggregate: Diamond centered at 0.40 with a 95% C I of 0.28 to 0.52.

- Non-clinical: Diamond centered at 0.41 with a 95% C I of 0.27 to 0.54.

3. Response Bias section.

- Aggregate: Diamond centered at negative 0.51 with a 95% C I of negative 0.80 to negative 0.22.

- Clinical: Wide diamond centered at negative 0.69 with a 95% C I of negative 1.56 to 0.17, crossing the zero line.

- Non-clinical: Diamond centered at negative 0.43 with a 95% C I of negative 0.74 to negative 0.11.

4. Perceptual Sensitivity section.

- Aggregate: Diamond centered at negative 0.17 with a 95% C I of negative 0.29 to negative 0.05.

- Clinical: Diamond centered at 0.25 with a 95% C I of negative 0.01 to 0.52, touching the zero line.

- Non-clinical: Diamond centered at negative 0.21 with a 95% C I of negative 0.33 to negative 0.09.

Navigate back to Figure 2..

### Table 3. Long description

The table is organized into two columns. The left column lists the category, and the right column lists the specific recommendations.

* Identifying AV-SDT s. Recommendations include the inclusion of signal detection in study identifying information such as title or keywords. It also suggests the explicit use of terminology for AV-SDT s, specifying they are signal detection tasks and the theoretical framework applied, such as reality monitoring or externalizing bias.

* Task parameters. Recommendations include specifying instructions given to participants, such as awareness of noise to signal frequency. It identifies features of trials including total number, ratio of noise to noise-and-signal, duration of trials, and sound presentation details. It also requires identifying study sounds, including noise type, voice content, frequencies, and volumes.

* Testing environment. Recommendations include identifying the equipment used and the physical location of the testing environment.

* Task outcomes. Recommendations focus on reporting signal detection outcomes, specifically including frequencies of hit rate, false alarm rate, perceptual sensitivity, and bias.

Navigate back to Table 3..

## References

[r1] Alderson-Day, B., Lima, C. F., Evans, S., Krishnan, S., Shanmugalingam, P., Fernyhough, C., & Scott, S. K. (2017). Distinct processing of ambiguous speech in people with non-clinical auditory verbal hallucinations. Brain, 140(9), 2475–2489. 10.1093/brain/awx206 29050393

[r2] Alganami, F., Varese, F., Wagstaff, G. F., & Bentall, R. P. (2017). Suggestibility and signal detection performance in hallucination-prone students. Cognitive Neuropsychiatry, 22(2), 159–174. 10.1080/13546805.2017.1294056 28253093

[r74] Alderson-Day, B., (2019). [Unpublished raw data on the correlation between hallucinatory experiences and performance on an auditory-verbal signal detection task]

[r3] Anderson, A., Hartley, S., & Bucci, S. (2021). A systematic review of the experimental induction of auditory perceptual experiences. Journal of Behavior Therapy and Experimental Psychiatry, 71, 101635. 10.1016/j.jbtep.2020.101635 33348277

[r4] Andreasen, N. C. (1984). *Scale for the Assessment of Positive Symptoms* [Data set]. 10.1037/t48377-000

[r5] Barkus, E., Smallman, R., Royle, N., Barkus, C., Lewis, S., & Rushe, T. (2011). Auditory false perceptions are mediated by psychosis risk factors. Cognitive Neuropsychiatry, 16(4), 289–302. 10.1080/13546805.2010.530472 21113827

[r6] Barkus, E., Stirling, J., Hopkins, R., McKie, S., & Lewis, S. (2007). Cognitive and neural processes in non-clinical auditory hallucinations. The British Journal of Psychiatry, 191(S51), s76–s81. 10.1192/bjp.191.51.s76 18055942

[r7] Bell, A., Toh, W. L., Moseley, P., Smailes, D., & Rossell, S. (unpublished). Cognitive mechanisms associated with auditory hallucinations within schizophrenia-spectrum disorders.

[r8] Bell, V., Halligan, P. W., & Ellis, H. D. (2006). The cardiff anomalous perceptions scale (CAPS): A new validated measure of anomalous perceptual experience. Schizophrenia Bulletin, 32(2), 366–377. 10.1093/schbul/sbj014 16237200 PMC2632213

[r9] Bentall, R. P., & Slade, P. D. (1985a). Reality testing and auditory hallucinations: A signal detection analysis. British Journal of Clinical Psychology, 24(3), 159–169. 10.1111/j.2044-8260.1985.tb01331.x 4052663

[r10] Bentall, R. P., & Slade, P. D. (1985b). Reliability of a scale measuring disposition towards hallucination: A brief report. Personality and Individual Differences, 6(4), 527–529. 10.1016/0191-8869(85)90151-5

[r11] Bristow, E., Tabraham, P., Smedley, N., Ward, T., & Peters, E. (2014). Jumping to perceptions and to conclusions: Specificity to hallucinations and delusions. Schizophrenia Research, 154(1–3), 68–72. 10.1016/j.schres.2014.02.004 24581551

[r12] Brookwell, M. L., Bentall, R. P., & Varese, F. (2013). Externalizing biases and hallucinations in source-monitoring, self-monitoring and signal detection studies: A meta-analytic review. Psychological Medicine, 43(12), 2465–2475. 10.1017/S0033291712002760 23282942

[r13] Chhabra, H., Selvaraj, S., Sreeraj, V. S., Damodharan, D., Shivakumar, V., Kumar, V., Narayanaswamy, J. C., & Venkatasubramanian, G. (2022). Functional near-infrared spectroscopy in schizophrenia patients with auditory verbal hallucinations: Preliminary observations. Asian Journal of Psychiatry, 73, 103127. 10.1016/j.ajp.2022.103127 35430497

[r14] Chhabra, H., Sowmya, S., Sreeraj, V. S., Kalmady, S. V., Shivakumar, V., Amaresha, A. C., Narayanaswamy, J. C., & Venkatasubramanian, G. (2016). Auditory false perception in schizophrenia: Development and validation of auditory signal detection task. Asian Journal of Psychiatry, 24, 23–27. 10.1016/j.ajp.2016.08.006 27931901

[r15] Cohen, J. (1992). Statistical power analysis. Current Directions in Psychological Science, 1(3), 98–101. 10.1111/1467-8721.ep10768783

[r16] de Boer, J. N., Linszen, M. M. J., de Vries, J., Schutte, M. J. L., Begemann, M. J. H., Heringa, S. M., Bohlken, M. M., Hugdahl, K., Aleman, A., Wijnen, F. N. K., & Sommer, I. E. C. (2019). Auditory hallucinations, top-down processing and language perception: A general population study. Psychological Medicine, 49(16), 2772–2780. 10.1017/S003329171800380X 30606279 PMC6877468

[r17] Downes, M. J., Brennan, M. L., Williams, H. C., & Dean, R. S. (2016). Development of a critical appraisal tool to assess the quality of cross-sectional studies (AXIS). BMJ Open, 6(12), e011458. 10.1136/bmjopen-2016-011458 PMC5168618 27932337

[r18] Duval, S., & Tweedie, R. (2000). Trim and fill: A simple funnel-plot-based method of testing and adjusting for publication bias in meta-analysis. Biometrics, 56(2), 455–463. 10.1111/j.0006-341x.2000.00455.x 10877304

[r19] Egger, M., Davey Smith, G., Schneider, M., & Minder, C. (1997). Bias in meta-analysis detected by a simple, graphical test. BMJ (Clinical Research Ed.), 315(7109), 629–634. 10.1136/bmj.315.7109.629 PMC2127453 9310563

[r20] Ghiani, A., Mann, D., & Brenner, E. (2024). Methods matter: Exploring how expectations influence common actions. iScience, 27(3), 109076. 10.1016/j.isci.2024.109076 38361615 PMC10867666

[r21] Hartung, J., & Knapp, G. (2001). On tests of the overall treatment effect in meta-analysis with normally distributed responses. Statistics in Medicine, 20(12), 1771–1782. 10.1002/sim.791 11406840

[r73] Higgins, J. P. T., & Thompson, S. G. (2002). Quantifying heterogeneity in a meta‐analysis. Statistics in Medicine, 21(11), 1539‑1558. Portico. 10.1002/sim.1186 12111919

[r22] Higgins, J. P. T., Thompson, S. G., Deeks, J. J., & Altman, D. G. (2003). Measuring inconsistency in meta-analyses. BMJ (Clinical Research Ed.), 327(7414), 557–560. 10.1136/bmj.327.7414.557 PMC192859 12958120

[r23] Higgins, J. P. T., Thompson, S. G., & Spiegelhalter, D. J. (2009). A re-evaluation of random-effects meta-analysis. Journal of the Royal Statistical Society, Series A, (Statistics in Society), 172(1), 137–159. 10.1111/j.1467-985X.2008.00552.x 19381330 PMC2667312

[r24] Hill, K., & Linden, D. E. J. (2013). Hallucinatory experiences in non-clinical populations. In R. Jardri, A. Cachia, P. Thomas, & D. Pins (Eds.), The neuroscience of hallucinations (pp. 21–41). Springer. 10.1007/978-1-4614-4121-2_2

[r25] Horga, G., Schatz, K. C., Abi-Dargham, A., & Peterson, B. S. (2014). Deficits in predictive coding underlie hallucinations in schizophrenia. Journal of Neuroscience, 34(24), 8072–8082. 10.1523/JNEUROSCI.0200-14.2014 24920613 PMC4051966

[r26] IntHout, J., Ioannidis, J. P. A., Rovers, M. M., & Goeman, J. J. (2016). Plea for routinely presenting prediction intervals in meta-analysis. BMJ Open, 6(7), e010247. 10.1136/bmjopen-2015-010247 PMC4947751 27406637

[r27] Kay, S. R., Opler, L. A., & Lindenmayer, J.-P. (1989). The positive and negative syndrome scale (PANSS): Rationale and standardisation. The British Journal of Psychiatry, 155(7), 59–65. 10.1192/S0007125000291514 2619982

[r28] Laloyaux, J., Hirnstein, M., Specht, K., Giersch, A., & Larøi, F. (2022). Eliciting false auditory perceptions using speech frequencies and semantic priming: A signal detection approach. Cognitive Neuropsychiatry, 27(4), 255–272. 10.1080/13546805.2022.2031945 35118930

[r29] Launay, G., & Slade, P. (1981). *Launay-Slade Hallucination Scale (LSHS).* 10.1037/t13160-000

[r30] Li, C.-S. R., Yang, Y.-Y., Chen, M.-C., Chen, W. J., & Liu, J.-L. (2003). Auditory discrimination in female adolescents varying in schizotypal features: Preliminary findings. Psychiatry and Clinical Neurosciences, 57(4), 391–397. 10.1046/j.1440-1819.2003.01137.x 12839520

[r31] Li, C. R., Chen, M., Yang, Y., Chen, M., & Tsay, P. (2002). Altered performance of schizophrenia patients in an auditory detection and discrimination task: Exploring the ‘self-monitoring’ model of hallucination. Schizophrenia Research, 55(1-2), 115–128. 10.1016/S0920-9964(01)00203-1 11955971

[r32] Lin, L., & Aloe, A. M. (2021). Evaluation of various estimators for standardized mean difference in meta-analysis. Statistics in Medicine, 40(2), 403–426. 10.1002/sim.8781 33180373 PMC7770064

[r33] Linszen, M. M. J., de Boer, J. N., Schutte, M. J. L., Begemann, M. J. H., de Vries, J., Koops, S., Blom, R. E., Bohlken, M. M., Heringa, S. M., Blom, J. D., & Sommer, I. E. C. (2022). Occurrence and phenomenology of hallucinations in the general population: A large online survey. Schizophrenia, 8(1), 41. 10.1038/s41537-022-00229-9 35853871 PMC9261095

[r34] Longden, E., Branitsky, A., Moskowitz, A., Berry, K., Bucci, S., & Varese, F. (2020). The relationship between dissociation and symptoms of psychosis: A meta-analysis. Schizophrenia Bulletin, 46(5), 1104–1113. 10.1093/schbul/sbaa037 32251520 PMC7505175

[r35] Losilla, J.-M., Oliveras, I., Marin-Garcia, J. A., & Vives, J. (2018). Three risk of bias tools lead to opposite conclusions in observational research synthesis. Journal of Clinical Epidemiology, 101, 61–72. 10.1016/j.jclinepi.2018.05.021 29864541

[r36] Lynn, S. K., & Barrett, L. F. (2014). “Utilizing” signal detection theory. Psychological Science, 25(9), 1663–1673. 10.1177/0956797614541991 25097061 PMC4304641

[r37] Ma, L.-L., Wang, Y.-Y., Yang, Z.-H., Huang, D., Weng, H., & Zeng, X.-T. (2020). Methodological quality (risk of bias) assessment tools for primary and secondary medical studies: What are they and which is better? Military Medical Research, 7(1), 7. 10.1186/s40779-020-00238-8 32111253 PMC7049186

[r38] Mason, O., Claridge, G., & Jackson, M. (1995). New scales for the assessment of schizotypy. Personality and Individual Differences, 18(1), 7–13. 10.1016/0191-8869(94)00132-C

[r39] McCarthy-Jones, S., & Fernyhough, C. (2011). The varieties of inner speech: Links between quality of inner speech and psychopathological variables in a sample of young adults. Consciousness and Cognition, 20(4), 1586–1593. 10.1016/j.concog.2011.08.005 21880511

[r40] Moher, D., Liberati, A., Tetzlaff, J., Altman, D. G., & PRISMA Group. (2009). Preferred reporting items for systematic reviews and meta-analyses: The PRISMA statement. PLoS Medicine, 6(7), e1000097. 10.1371/journal.pmed.1000097 19621072 PMC2707599

[r41] Montemayor, C., & Haladjian, H. H. (2017). Perception and cognition are largely independent, but still affect each other in systematic ways: Arguments from evolution and the consciousness-attention dissociation. Frontiers in Psychology, 8, 40. 10.3389/fpsyg.2017.00040 28174551 PMC5258763

[r42] Morrison, A. P., Wells, A., & Nothard, S. (2000). Cognitive factors in predisposition to auditory and visual hallucinations. British Journal of Clinical Psychology, 39(1), 67–78. 10.1348/014466500163112 10789029

[r43] Moseley, P., Alderson-Day, B., Common, S., Dodgson, G., Lee, R., Mitrenga, K., Moffatt, J., & Fernyhough, C. (2022). Continuities and discontinuities in the cognitive mechanisms associated with clinical and nonclinical auditory verbal hallucinations. Clinical Psychological Science, 10(4), 752–766. 10.1177/21677026211059802 35846173 PMC9280701

[r44] Moseley, P., Aleman, A., Allen, P., Bell, V., Bless, J., Bortolon, C., Cella, M., Garrison, J., Hugdahl, K., Kozáková, E., Larøi, F., Moffatt, J., Say, N., Smailes, D., Suzuki, M., Toh, W. L., Woodward, T., Zaytseva, Y., … Fernyhough, C (2021). Correlates of hallucinatory experiences in the general population: An international multisite replication study. Psychological Science, 32(7), 1024–1037. 10.1177/0956797620985832 34087077 PMC8641136

[r45] Moseley, P., Fernyhough, C., & Ellison, A. (2013). Auditory verbal hallucinations as atypical inner speech monitoring, and the potential of neurostimulation as a treatment option. Neuroscience and Biobehavioral Reviews, 37(10), 2794–2805. 10.1016/j.neubiorev.2013.10.001 24125858 PMC3870271

[r46] Moseley, P., Smailes, D., Ellison, A., & Fernyhough, C. (2016). The effect of auditory verbal imagery on signal detection in hallucination-prone individuals. Cognition, 146, 206–216. 10.1016/j.cognition.2015.09.015 26435050 PMC4675095

[r47] Nair, A. S. (2019). Publication bias—Importance of studies with negative results! Indian Journal of Anaesthesia, 63(6), 505–507. 10.4103/ija.IJA_142_19 31263309 PMC6573059

[r48] Nazimek, J. M., Hunter, M. D., & Woodruff, P. W. R. (2012). Auditory hallucinations: Expectation-perception model. Medical Hypotheses, 78(6), 802–810. 10.1016/j.mehy.2012.03.014 22520337

[r49] Os, J. v., Linscott, R. J., Myin-Germeys, I., Delespaul, P., & Krabbendam, L. (2009). A systematic review and meta-analysis of the psychosis continuum: Evidence for a psychosis proneness–persistence–impairment model of psychotic disorder. Psychological Medicine, 39(2), 179–195. 10.1017/S0033291708003814 18606047

[r50] Ouzzani, M., Hammady, H., Fedorowicz, Z., & Elmagarmid, A. (2016). Rayyan—A web and mobile app for systematic reviews. Systematic Reviews, 5(1), 210. 10.1186/s13643-016-0384-4 27919275 PMC5139140

[r51] Powers, A. R., Kelley, M., & Corlett, P. R. (2016). Hallucinations as top-down effects on perception. Biological Psychiatry: Cognitive Neuroscience and Neuroimaging, 1(5), 393–400. 10.1016/j.bpsc.2016.04.003 28626813 PMC5469545

[r52] Powers, A. R., Mathys, C., & Corlett, P. R. (2017). Pavlovian conditioning–induced hallucinations result from overweighting of perceptual priors. Science, 357(6351), 596–600. 10.1126/science.aan3458 28798131 PMC5802347

[r53] Rankin, P. M., & O’Carroll, P. J. (1995). Reality discrimination, reality monitoring and disposition towards hallucination. British Journal of Clinical Psychology, 34(4), 517–528. 10.1111/j.2044-8260.1995.tb01486.x 8563659

[r54] Riley, R. D., Higgins, J. P. T., & Deeks, J. J. (2011). Interpretation of random effects meta-analyses. BMJ, 342, d549. 10.1136/bmj.d549 21310794

[r55] Rodriguez-Sanchez, J., Oloye, H., Martin, I. M., & Hauke, D. J. (2023). Evidence for a primary prior deficit as a mechanism of auditory hallucinations. The Journal of Neuroscience, 43(50), 8579–8581. 10.1523/JNEUROSCI.1601-23.2023 38092525 PMC10727187

[r56] Rollins, C. P. E., Garrison, J. R., Simons, J. S., Rowe, J. B., O’Callaghan, C., Murray, G. K., & Suckling, J. (2019). Meta-analytic evidence for the plurality of mechanisms in transdiagnostic structural MRI studies of hallucination status. eClinicalMedicine, 8, 57–71. 10.1016/j.eclinm.2019.01.012 31193632 PMC6537703

[r57] Romme, M. A., & Escher, A. D. (1989). Hearing voices. Schizophrenia Bulletin, 15(2), 209–216. 10.1093/schbul/15.2.209 2749184

[r58] Shenyan, O., Haye, L., Milne, G. A., Lisi, M., Greenwood, J. A., Skipper, J. I., & Dekker, T. M. (2025). Reduced susceptibility to experimentally-induced complex visual hallucinations with age. Cortex, 191, 188–204. 10.1016/j.cortex.2025.08.001 40885086

[r59] Smailes, D., Meins, E., & Fernyhough, C. (2014). The impact of negative affect on reality discrimination. Journal of Behavior Therapy and Experimental Psychiatry, 45(3), 389–395. 10.1016/j.jbtep.2014.04.001 24809623

[r60] Smailes, D., Meins, E., & Fernyhough, C. (2015). Associations between intrusive thoughts, reality discrimination and hallucination-proneness in healthy young adults. Cognitive Neuropsychiatry, 20(1), 72–80. 10.1080/13546805.2014.973487 25345759

[r62] Stokes, D. (2012). Perceiving and desiring: A new look at the cognitive penetrability of experience. Philosophical Studies, 158(3), 477–492. 10.1007/s11098-010-9688-8

[r63] Strawson, W. H., Wang, H.-T., Quadt, L., Sherman, M., Larsson, D. E. O., Davies, G., Mckeown, B. L. A., Silva, M., Fielding-Smith, S., Jones, A.-M., Hayward, M., Smallwood, J., Critchley, H. D., & Garfinkel, S. N. (2022). Voice hearing in borderline personality disorder across perceptual, subjective, and neural dimensions. International Journal of Neuropsychopharmacology, 25(5), 375–386. 10.1093/ijnp/pyab093 34940826 PMC9154289

[r64] Turner, R. M., Bird, S. M., & Higgins, J. P. T. (2013). The impact of study size on meta-analyses: Examination of underpowered studies in Cochrane reviews. PLoS One, 8(3), e59202. 10.1371/journal.pone.0059202 23544056 PMC3609745

[r65] van Os, J., Hanssen, M., Bijl, R. V., & Ravelli, A. (2000). Strauss (1969) revisited: A psychosis continuum in the general population? Schizophrenia Research, 45(1–2), 11–20. 10.1016/s0920-9964(99)00224-8 10978868

[r66] Varese, F., Barkus, E., & Bentall, R. P. (2011). Dissociative and metacognitive factors in hallucination-proneness when controlling for comorbid symptoms. Cognitive Neuropsychiatry, 16(3), 193–217. 10.1080/13546805.2010.495244 20694861

[r67] Varese, F., Barkus, E., & Bentall, R. P. (2012). Dissociation mediates the relationship between childhood trauma and hallucination-proneness. Psychological Medicine, 42(5), 1025–1036. 10.1017/S0033291711001826 21896238

[r68] Vercammen, A., de Haan, E. H. F., & Aleman, A. (2008). Hearing a voice in the noise: Auditory hallucinations and speech perception. Psychological Medicine, 38(8), 1177–1184. 10.1017/S0033291707002437 18076771

[r69] Vetter, P., & Newen, A. (2014). Varieties of cognitive penetration in visual perception. Consciousness and Cognition, 27, 62–75. 10.1016/j.concog.2014.04.007 24836978

[r70] Waters, F., Allen, P., Aleman, A., Fernyhough, C., Woodward, T. S., Badcock, J. C., Barkus, E., Johns, L., Varese, F., Menon, M., Vercammen, A., & Larøi, F. (2012). Auditory hallucinations in schizophrenia and nonschizophrenia populations: A review and integrated model of cognitive mechanisms. Schizophrenia Bulletin, 38(4), 683–693. 10.1093/schbul/sbs045 22446568 PMC3406530

[r71] Waters, F., & Fernyhough, C. (2017). Hallucinations: A systematic review of points of similarity and difference across diagnostic classes. Schizophrenia Bulletin, 43(1), 32–43. 10.1093/schbul/sbw132 27872259 PMC5216859

[r72] Waters, F., & Fernyhough, C. (2019). Auditory hallucinations: Does a continuum of severity entail continuity in mechanism? Schizophrenia Bulletin, 45(4), 717–719. 10.1093/schbul/sbz002 30753702 PMC6581142

