# Supplementary material for: Hallucinations and auditory-verbal signal detection studies: A meta-analytic review
Source: Psychol Med. 2026 Aug 10;56:e254. doi: 10.1017/S0033291726105339 (PMC13468945; doi:10.1017/S0033291726105339)
Supplement: Supplementary file 1 [file S0033291726105339sup001.docx]

**Hallucinations and auditory-verbal signal detection studies: a meta-analytic review**

**Supplementary Materials**

The standardised data extraction form used within this project is available here: <https://osf.io/mt9vu/files/gdk9y>

Comparison of reviewers decision agreement after title and abstract screening is available here: <https://osf.io/mt9vu/files/eu2bp>

**Table 1**

Manipulation guides for online database searches

| ***No.*** | ***Databases (Total 3)*** | ***Explanation*** |
| --- | --- | --- |
| **1** | **PubMed** | 1. Perform PubMed search.  2. Click on Send To, choose Citation Manager.  3. Select ‘All results’  4. Click Create File.  5. Move PubMed file to a suitable directory. |
| **2** | **Scopus** | 1. Perform Scopus search.  2. Select all articles.  3. Click on Export.  4. On the new interface, choose RIS format.  5. At Choose the Information to export, choose All available information.  6. Click on Export.  7. Move Scopus file to a suitable directory. |
| **3** | **Web of Science (WoS)** | 1. Perform WoS search.  2. Select all articles.  3. In the Save to panel, click on EndNote Desktop  4. Move WoS file to a suitable directory. |

**Table 2**

Detailed search strategy for database searches

| *No.* | *Databases (Total 3)* | *Search Terms* | *Results*  *Total =* |
| --- | --- | --- | --- |
| **1** | **PubMed** | **(((("hallucina*"[Title/Abstract]) AND ("voice*"[Title/Abstract]) OR "voice-hearing"[TITLE/ABSTRACT]) OR ("psychosis*"[Title/Abstract] OR "schizo*"[Title/Abstract])) AND (("signal detection"[Title/Abstract]) OR ("auditory"[Title/Abstract] OR "auditory signal"[Title/Abstract]) OR ("perceptual bias"[Title/Abstract] OR "signal sensitivity"[Title/Abstract] OR "decision making"[Title/Abstract] OR "externalising bias"[Title/Abstract] OR "reality discrimination"[Title/Abstract]) OR "source monitoring"[Title/Abstract])) AND (1985:2022[pdat]) AND ("subject" OR "participant")**  (("hallucina*"[Title/Abstract] AND "voice*"[Title/Abstract]) OR "voice-hearing"[Title/Abstract] OR ("psychosis*"[Title/Abstract] OR "schizo*"[Title/Abstract])) AND ("signal detection"[Title/Abstract] OR ("auditory"[Title/Abstract] OR "auditory signal"[Title/Abstract]) OR ("perceptual bias"[Title/Abstract] OR "signal sensitivity"[Title/Abstract] OR "decision making"[Title/Abstract] OR "externalising bias"[Title/Abstract] OR "reality discrimination"[Title/Abstract]) OR "source monitoring"[Title/Abstract]) AND 1985/01/01:2022/12/31[Date - Publication] AND ("subject"[All Fields] OR "trials"[All Fields] OR "participant"[All Fields]) | **330** |
| **2** | **Scopus** | TITLE-ABS-KEY((("Hallucinat*") AND ("voice*" OR "voice-hearing") OR ("psychosis*" OR "schizo*") AND ("signal detection") OR ("auditory" OR "auditory signal") OR ("perceptual bias" OR "signal sensitivity" OR " decision making" OR "exernalising bias" OR "reality discrimination" OR "source monitoring"))) AND ALL (“subject” or “participant”) AND PUBYEAR > 1984 | **2050** |

**Table 2** continued

| *No.* | *Databases (Total 3)* | *Search Terms* | *Results*  *Total =* |
| --- | --- | --- | --- |
| **3** | **Web of Science** | **((((((AB=(Hallucina*))) AND (AB=(voice*)) OR AB=(voice-hearing))) OR (AB=(psychosis*)) OR (AB=(psycho*)) OR AB=(schizo*)))**  AND  **(((((((AB=(signal detection))) OR (AB=(auditory)) OR AB=(auditory signal))) OR (AB=(perceptual bias)) OR AB=(signal sensitivity)) OR AB=(decision making)) OR AB=(externalising bias)) OR AB=(reality discrimination) OR AB =(source monitoring) AND (ALL=(participant) OR ALL=(subject))**  AND  **((((((TI=(Hallucina*))) AND (TI=(voice*)) OR TI=(voice-hearing))) OR (TI=(psychosis*)) OR (TI=(psycho*)) OR TI=(schizo*)))**  AND  **(((((((TI=(signal detection))) OR (TI=(auditory)) OR TI=(auditory signal))) OR (TI=(perceptual bias)) OR TI=(signal sensitivity)) OR TI=(decision making)) OR TI=(externalising bias)) OR TI=(reality discrimination) OR TI =(source monitoring) AND (ALL=(participant) OR ALL=(subject))**  Date range – 01.01.1985-19-07-2022  Link to search -  <https://www.webofscience.com/wos/woscc/summary/5077b0c9-a4b5-423f-b55e-91dc9e651976-4303d1e6/relevance/1> | **1755** |
|  | | **Total included before screening-** | **4135** |

**Table 3**

PICOS table of study inclusion criteria

| **P**opulations | **I**nterest | **C**ontrol/**C**omparison | **O**utcome Measures | **S**tudy Types |
| --- | --- | --- | --- | --- |
| Human Studies | Examine the relationship between hallucinations or hallucination-proneness and signal detection using auditory signal detection tasks | In studies reporting between-group designs, clearly defined comparisons between hallucination prone and comparable non-prone participant groups (this review did not discriminate between the modalities of hallucinations, i.e. all clinical patients (patients with a psychiatric diagnosis) with hallucinations vs non-hallucinating clinical patients; non-clinical psychotic-like experiences vs. control participants were eligible for inclusion | Perceptual sensitivity, Response Bias, and False Alarm Rates | All quantitative, between-group or correlational design studies |

**Table 4** Table of effect sizes of individual studies included within these meta-analyses

| Outcome | Study | Sample  (Clinical vs non-clinical) | Measure | Low Hallucination Group | | | High Hallucination Group | | | Cohens D | Hedges G (*SE*) |
| --- | --- | --- | --- | --- | --- | --- | --- | --- | --- | --- | --- |
|  |  |  |  | n | Mean | SD | n | Mean | SD |  |  |
| Hit Rate | Bell et al. (under review) | Clinical |  | 20 | 0.68 | 0.19 | 19 | 0.68 | 0.22 | 0.01 (0.07) | 0.01 (0.43) |
| False Alarm Rate | Barkus et al. (2011)  Bell et al. (under review) | Clinical  Clinical |  | 25  20 | 3.28  0.34 | 3.79  0.29 | 30  19 | 5.07  0.40 | 5.36  0.28 | 0.38 (1.28)  0.20 (0.09) | 0.37 (0.37)  0.20 (0.43) |
|  | Alganami et al. (2017) | Non-clinical |  | 31 | 1.96 | 1.45 | 31 | 3.55 | 2.74 | 0.73 (0.56) | 0.72 (0.35) |
|  | Moseley et al. (2022) | Non-clinical |  | 28 | 11.38 | 23.30 | 26 | 20.43 | 22.22 | 0.40 (6.21) | 0.39 (0.37) |
| Perceptual sensitivity | Bentall & Slade (1985)  Bell et al. (under review) | Clinical  Clinical | *P*(*A*)  *d′* | 10  20 | 0.77  1.09 | 0.12  0.53 | 10  19 | 0.77  0.91 | 0.11  0.46 | 0.00 (0.05)  0.35 (0.16) | 0.00 (0.58)  0.35 (0.43) |
|  | Varese et al. (2012) | Clinical | *d′* | 16 | 1.40 | 0.44 | 15 | 1.07 | 0.44 | 0.75 (0.16) | 0.73 (0.49) |
|  | Alganami et al. (2017) | Non-clinical | *d′* | 31 | 1.64 | 0.39 | 31 | 1.51 | 0.38 | -0.34 (0.10) | -0.33 (0.35) |
|  | Bentall & Slade (1985) | Non-clinical | *P*(*A*) | 10 | 0.87 | 0.03 | 10 | 0.87 | 0.03 | 0.00 (0.01) | 0.00 (0.58) |
|  | Moseley et al. (2022) | Non-clinical | *d′* | 28 | 2.27 | 0.59 | 26 | 1.95 | 0.58 | -0.55 (0.16) | -0.54 (0.38) |
|  | Rankin & O’Carroll (1995) | Non-clinical | *P*(*A*) | 15 | 0.63 | 0.16 | 15 | 0.67 | 0.12 | 0.28 (0.05) | 0.28 (0.49) |
|  | Varese et al. (2011) | Non-clinical | *d′* | 33 | 1.16 | 0.50 | 34 | 1.11 | 0.44 | -0.11 (0.11) | -0.11 (0.34) |
| Response bias | Barkus et al. (2011)  Bell et al. (under review) | Clinical  Clinical | β  β | 25  20 | 0.30  2.25 | 0.54  3.19 | 30  19 | 0.10  1.24 | 0.54  0.99 | -0.37 (0.15)  -0.42 (0.77) | -0.37 (0.37)  -0.41 (0.43) |
|  | Bentall & Slade (1985) | Clinical | β | 10 | 3.78 | 0.86 | 10 | 2.63 | 0.95 | -1.27 (0.41) | -1.22 (0.61) |
|  | Bristow et al (2014) | Clinical | β | 51 | 3.27 | 1.24 | 47 | 2.75 | 1.48 | -0.38 (0.28) | -0.38 (0.28) |
|  | Varese et al. (2012) | Clinical | β | 16 | 0.56 | 0.35 | 15 | 0.19 | 0.38 | -1.01 (0.13) | -0.99 (0.49) |
|  | Alganami et al. (2017) | Non-clinical | β | 31 | 0.59 | 0.24 | 31 | 0.40 | 0.35 | -0.63 (0.08) | -0.63 (0.35) |
|  | Bentall & Slade (1985) | Non-clinical | β | 10 | 3.77 | 0.50 | 10 | 2.69 | 0.85 | -1.55 (0.31) | -1.48 (0.62) |
|  | Moseley et al. (2022) | Non-clinical | β | 28 | 4.99 | 3.60 | 26 | 2.79 | 2.97 | -0.67 (0.90) | -0.65 (0.38) |
|  | Rankin & O’Carroll (1995) | Non-clinical | β” | 15 | 2.60 | 0.19 | 15 | 1.86 | 0.60 | -1.66 (0.16) | -1.62 (0.53) |
|  | Varese et al. (2011) | Non-clinical | β | 33 | 0.22 | 0.37 | 34 | 0.14 | 0.35 | -0.22 (0.09) | -0.22 (0.34) |

**Table 5** Table of effect sizes of individual correlational studies included within these meta-analyses

| SD Outcome | Study | Sample  (Clinical vs non-clinical) | Measure | |  |  | | Association between HP and Analysis | | | |
| --- | --- | --- | --- | --- | --- | --- | --- | --- | --- | --- | --- |
|  |  |  |  | *N* | | *R (df)* | Lower Limit | | Upper Limit | Cohens D | Hedges G (*SE)* |
| False Alarm Rate | Alderson-Day (2019) | Non-clinical |  | | 95 | .18 (94) |  | |  | 0.36 | 0.36 (0.15) |
|  | Barkus et al. (2007) | Non-clinical |  | | 62 | .31 (61) |  | |  | 0.64 | 0.63 (0.19) |
|  | Moseley et al. (2021) | Non-clinical |  |  | 583 | .14 (581) | .06 | | .22 | 0.28 | 0.28 (0.06) |
|  | Smailes et al. (2015) | Non-clinical |  |  | 153 | .25 (152) |  | |  | 0.51 | 0.51 (0.12) |
| Hit Rate | Alderson-Day (2019) | Non-clinical |  | | 95 | .14 (94) |  | |  | 0.28 | 0.28 (0.15) |
|  | Barkus et al. (2007) | Non-clinical |  | | 62 | .36 (61) |  | |  | 0.78 | 0.77 (0.19) |
|  | Moseley et al. (2021) | Non-clinical |  | | 583 | .18 (581) | .10 | | .26 | 0.37 | 0.37 (0.06) |
|  | Smailes et al. (2015) | Non-clinical |  | | 153 | .24 (152) |  | |  | 0.49 | 0.49 (0.12) |
| Perceptual sensitivity | Strawson et al. (2021) | Clinical | *d′* | | 22 | .18 (19) | -.28 | | .57 | 0.37 | 0.35 (0.31) |
|  | Alderson-Day (2019) | Non-clinical | *d′* | | 95 | -.15 (94) |  | |  | -0.30 | -0.30 (0.15) |
|  | Barkus et al. (2007) | Non-clinical | *d′* | | 62 | -.09 (61) |  | |  | -0.17 | -0.17 (0.18) |
|  | Moseley et al. (2021) | Non-clinical | *d′* | | 583 | -.05(581) | -.13 | | .03 | -0.10 | -0.10 (0.06) |
|  | Smailes et al. (2015) | Non-clinical | *d′* | | 153 | -.18 (152) |  | |  | -0.37 | -0.37 (0.12) |
| Response bias | Strawson et al. (2021) | Clinical | *C* | | 22 | -.65 (19) | -.85 | | -.31 | -1.71 | -1.65 (0.39) |
|  | Alderson-Day (2019) | Non-clinical | β | | 95 | -.19 (94) |  | |  | -0.39 | -0.38 (0.15) |
|  | Barkus et al. (2007) | Non-clinical | β” | | 62 | .12 (61) |  | |  | 0.24 | 0.24 (0.18) |
|  | Moseley et al. (2021) | Non-clinical | β | | 583 | -.17 (581) | -.25 | | -.09 | -0.35 | -0.34 (0.06) |
|  | Smailes et al. (2015) | Non-clinical | β | | 153 | -.26 (152) |  | |  | -0.54 | -0.54 (0.12) |

**Table 6** AV-SDTs design extracted from included published studies

|  | Author (Year) | How was the task described in the paper? | How was the voice described to participants? | Were participants told about ratio of noise to noise and signal trials? | If yes, what was this described as? | Were practise runs included? | Did participants receive feedback on their practise performance? |
| --- | --- | --- | --- | --- | --- | --- | --- |
| (1) | Alganami et al. (2017) | ASDT |  | Yes | 30% of trials a voice will be present | Yes | Not clear |
| (2) | Barkus et al. (2007) | ASDT | Androgynous | Not clear |  | Not clear | Not clear |
| (3) | Barkus et al. (2011) | ASDT | Speech | Not clear |  | Not clear | Not clear |
| (4) | Bentall & Slade (1985) | Not clear | Given example | Yes | ‘Sometimes’ | Yes | Not clear |
| (5) | Bristow et al. (2014) | Not clear |  | Not clear |  | Yes | Yes |
| (6) | Moseley et al. (2022) | ASDT | Speech | No |  | Not clear | Not clear |
| (7) | Moseley et al. (2021) | ASDT |  | No |  | Not clear | Not clear |
| (8) | Rankin & O’Carroll (1995) | SDM / Listening Task | Given example | Yes | ‘Some’ | Yes | Not clear |
| (9) | Smailes et al. (2015) | SDT |  | Not clear |  | Not clear | Not clear |
| (10) | Strawson et al. (2021) | Not clear | Not clear | Not clear |  | Not clear | Not clear |
| (11) | Varese et al. (2011) | Not clear | Voice | Not clear |  | Not clear | Not clear |
| (12) | Varese et al. (2012) | ASDT | Hearing test | Not clear |  | Not clear | Not clear |

*Notes:* ASDT = Auditory Signal Detection Task; SDM = Signal Detection Measure; SDT = Signal Detection Task

|  | Required responses | Number of task runs per session | Number of task blocks per run | Number of task trials per block | Number of noise only trials per block | Number of noise and signal trials per block | Type of noise | Language of stimuli | Length of time noise presented for each trial (sec) | Delay before stimuli presented in noise (sec) | Length of time stimuli presented for within noise (sec) | Length of time to respond (sec) |
| --- | --- | --- | --- | --- | --- | --- | --- | --- | --- | --- | --- | --- |
| (1) | Yes or No | 3 | 1 | 60 | 24 | 36 | WN | Not clear | 5 | Not clear | 1 | Not clear |
| (2) | Yes or No | 3 | 1 | 60 | 24 | 36 | WN | English | 5 | 2 | 1 | 3 |
| (3) | Yes or No | 3 | 1 | 60 | 24 | 36 | WN | English | 5 | 2 | 1 | 3 |
| (4) | Yes or No | 1 | 2 | 50 | 25 | 25 | WN | English | 5 | 3 | Not clear | 8 |
| (5) | Yes or No | 1 | 1 | 40 | 20 | 20 | WN | English | 5 | 3 | Not clear | 8 |
| (6) | Yes or No | 1 | 1 | 80 | 32 | 48 | PN | Not clear | 3.5 | Not clear | 1.5 | Not clear |
| (7) | Yes or No | 1 | 1 | 72 | 36 | 36 | WN | English | 3.5 | 1 | 1.5 | Not clear |
| (8) | 5-point certainty scale | 1 | 1 | 100 | 50 | 50 | WN | English | 5 | 3 |  | 5 |
| (9) | Yes or No |  | 1 | 60 | 26 | 34 | WN | Not clear | 5 | 2 | 1 | 3 |
| (10) | Present or Not Present | 1 | 5 | 40 | 20 | 20 | WN | English | 1.5 | R | 0.2 | SP |
| (11) | Yes or No | 1 | 2 | 60 | 24 | 36 | WN | English | 5 | 2 | 1 | 3 |
| (12) | Yes or No | 1 | 2 | 120 | 48 | 72 | WN | English | 5 | 2 | 1 | 3 |

**Table 6** *continued*

*Notes:* WN = White Noise; PN = Pink Noise; R = Random; SP = Self-pace

**Figure 1**

Forest plot of the aggregate and non-clinical analyses for the association between hallucinatory experiences and AV-SDT false alarm rate. CI = Confidence interval

**Figure 2**

Forest plot of the aggregate and non-clinical analyses for the association between hallucinatory experiences and AV-SDT hit rate. CI = Confidence interval

**Figure 3**

Forest plot of the aggregate and subgroup analyses (clinical and non-clinical) for the association between hallucinatory experiences and perceptual sensitivity. CI = Confidence interval

**Figure 4**

Forest plot of the aggregate and subgroup analyses (clinical and non-clinical) for the association between hallucinatory experiences and response bias. CI = Confidence interval

**Table 7**

Results of Egger's Test for funnel plot asymmetry for study models

| Outcome | HE Model | *Model Effects* | | |
| --- | --- | --- | --- | --- |
|  |  | Model intercept (95% CI) | t(df) | *p* |
| False Alarm Rate | Aggregate | 0.31 (0.11, 0.51) | 1.16 (6) | 0.290 |
|  | Non-clinical | 0.26 (0.07, 0.46) | 1.81 (5) | 0.130 |
| Hit Rate | Aggregate | 0.42 (0.04, 0.80) | -0.07 (4) | 0.945 |
|  | Non-clinical | 0.33 (-0.03, 0.69) | 0.75 (3) | 0.507 |
| Perceptual Sensitivity | Aggregate | -0.26 (-0.47, -0.05) | 1.25 (11) | 0.238 |
|  | Clinical | 0.76 (-0.16, 1.68) | 2.45 (2) | 0.134 |
|  | Non-clinical | -0.21 (-0.42, 0.01) | 0.07 (7) | 0.950 |
| Response Bias | Aggregate | -0.08 (-0.57, 0.43) | 2.10 (13) | 0.055 |
|  | Clinical | -0.35 (-5.05, 4.35) | -0.24 (3) | 0.824 |
|  | Non-clinical | -0.06 (-0.56 , 0.44) | -2.05 (8) | 0.074 |

**Table 8**

Results of Leave-one-out sensitivity analysis for study models where omission of one study substantially influenced study effect (>10% change)

Note: Main aggregate effects are listed in bold

**Table 9**

Axis quality assessment ratings of published studies

| Author, Year | Axis Items | | | | | | | | | | | | | | | | | | | | **Reporting Quality** | Study Design | Possible biases | Overall % Yes | Quality Rank |
| --- | --- | --- | --- | --- | --- | --- | --- | --- | --- | --- | --- | --- | --- | --- | --- | --- | --- | --- | --- | --- | --- | --- | --- | --- | --- |
|  | 1 | 2 | 3 | 4 | 5 | 6 | 7 | 8 | 9 | 10 | 11 | 12 | 13 | 14 | 15 | 16 | 17 | 18 | 19 | 20 |  |  |  |  |  |
| Alganami et al. (2017) | Y | Y | N | Y | N | Y | Y | Y | Y | N | N | Y | D | D | Y | D | Y | Y | N | Y | 4 | 4 | 4 | 60 | Medium |
| Barkus et al. (2007) | Y | Y | N | Y | N | Y | D | Y | D | Y | Y | Y | D | D | Y | Y | Y | Y | N | Y | 7 | 4 | 2 | 65 | Medium |
| Barkus et al. (2011) | Y | Y | N | Y | Y | D | D | Y | Y | N | Y | Y | D | D | D | Y | Y | Y | D | Y | 6 | 5 | 1 | 60 | Medium |
| Bentall & Slade (1985) | Y | Y | N | Y | N | N | D | Y | D | Y | D | Y | N | D | D | D | Y | N | D | D | 4 | 3 | 0 | 35 | Low |
|  | Y | Y | N | Y | Y | Y | D | Y | Y | Y | D | Y | N | D | D | D | Y | N | D | D | 4 | 4 | 2 | 50 | Low |
| Bristow et al. (2014) | Y | Y | N | Y | Y | Y | D | Y | D | Y | D | Y | D | D | D | Y | Y | Y | N | D | 6 | 4 | 1 | 55 | Low |
| Moseley et al. (2022) | Y | Y | Y | Y | N | Y | D | Y | Y | Y | Y | Y | D | D | D | Y | Y | Y | N | Y | 7 | 5 | 2 | 70 | Medium |
| Moseley et al. (2021) | Y | Y | Y | Y | Y | Y | Y | Y | Y | Y | Y | Y | D | N | Y | Y | Y | Y | N | Y | 7 | 6 | 4 | 85 | High |
| Rankin & O’Carroll (1995) | Y | Y | N | Y | N | Y | D | Y | D | N | N | Y | N | Y | D | D | Y | N | D | D | 3 | 3 | 2 | 40 | Low |
| Smailes et al. (2015) | D | Y | N | Y | N | D | D | Y | Y | Y | Y | Y | D | D | Y | D | Y | Y | D | Y | 5 | 4 | 2 | 55 | Low |
| Strawson et al. (2021) | Y | Y | N | Y | Y | Y | D | Y | D | N | Y | Y | N | Y | D | Y | Y | Y | N | Y | 6 | 5 | 2 | 65 | Medium |
| Varese et al. (2011) | Y | Y | N | Y | N | Y | D | Y | Y | Y | Y | Y | D | D | Y | D | Y | Y | D | Y | 6 | 4 | 3 | 65 | Medium |
| Varese et al. (2012) | Y | Y | N | Y | Y | Y | D | Y | Y | Y | Y | Y | D | D | Y | Y | Y | Y | N | Y | 7 | 5 | 3 | 75 | Medium |
| Mean subscale score across all studies | | | | | | | | | | | | | | | | | | | | | 5.54 | 4.31 | 2.15 |  |  |

**Axis Quality Assessment Items**

1. Were the aims/ objectives of the study clear?
2. Was the study design appropriate for the stated aim?
3. Was the sample justified?
4. Was the target population clearly defined?
5. Was the sample frame taken from an appropriate population base so that it closely represented the target population under investigation?
6. Was the selection process likely to select subjects / participants that were representative of the target / reference population under investigation?
7. Were measures undertaken to address and categorise non-responders?
8. Were the risk factor and outcome variables measured appropriate to the aims of the study?
9. Were the risk factor and outcome variables measured correctly using instruments/measurements that had been trialled, piloted or published previously?
10. Is it clear what was used to determine statistical significance and/or precision estimates? (e.g. p-values, confidence intervals)
11. Were the methods (including statistical methods) sufficiently described to enable them to be repeated?
12. Were the basic data adequately described?
13. Does the response rate raise concerns about non-response bias?
14. If appropriate, was information about non-responders described?
15. Were the results internally consistent?
16. Were the results presented for all the analyses described in the methods?
17. Were the authors' discussions and conclusions justified by the results?
18. Were the limitations of the study discussed?
19. Were there any funding sources or conflicts of interest that may affect the authors' interpretation of results?
20. Was ethical approval or consent of participants attained?

**Table 10**

Results from the AXIS quality assessment of published hallucinations literature containing AV-SDTs

*Note:* Items from the AXIS tool were grouped together according to items relating to A) reporting quality, B) study design, and C) possible biases
